# Impact of Ureteral Length on Urological Complications and Patient Survival After Kidney Transplantation

**DOI:** 10.5812/numonthly.10881

**Published:** 2013-08-03

**Authors:** Majid Ali-Asgari, Farid Dadkhah, Alireza Ghadian, Mohammad Hossein Nourbala

**Affiliations:** 1 Department of Urology, Shaheed Modarres Hospital, Shaheed Beheshti University of Medical Sciences, Tehran, IR Iran; 2 Nephrology and Urology Research Center, Baqiyatallah University of Medical Sciences, Tehran, IR Iran

**Keywords:** Ureter, Kidney Transplantation, Complications

## Abstract

**Background:**

Urologic complications are of the most important complications after kidney transplantation which increases mortality and morbidity significantly.

**Objectives:**

We designed this study to evaluate the association between ureteral length and postoperative complications.

**Patients and Methods:**

We recorded the length of the transplanted ureter during the operation. Ureter-to-bladder anastomosis was performed using modified Lich-Gregoir method on the ureteral stent. Complications like urine leakage and increased creatinine were evaluated. We used both univariate and multivariate analyses and survival analysis according lengths of ureter. It means that the main variable is ureteral length and other variables are studied based on it.

**Result:**

A total of 395 patients with the mean age of 37 years (range, 18 to 68 years) were enrolled in the study, twenty six graft lost during the follow-up period. The Mean age of recipients was 37 ± 13 years. Urinary stenosis was seen in 6 patients (1.5%) and urinary leakage in 4 (1%) patients. The complication rate was not significantly different between these groups (P = 0.67). We found that there were no significant difference among complication (P = 0.25), hospitalization (P = 0.31) and survival (P = 0.84) at 5.5 cm length cut off.

**Conclusions:**

The length of transplanted ureter does not affect the postoperative urologic complications (including urinary fistula and ureter-to-bladder anastomosis stricture), and it seems that decreased rate of complication frequency during the recent years is due to technical improvement, surgeon’s skillfulness and development in use of immunosuppressant’s postoperatively.

## 1. Background

Urological complications remain an important source of morbidity and occasionally mortality, after renal transplantation (1, 2). Urinary anastomotic complications following renal transplantation cause significant patient morbidity (3, 4). Urologic complications after renal transplantation may cause significant morbidity which seriously affects graft outcomes. The incidence of these complications has been reported to be 6.5% to 20%. Many donor, recipient, and post transplantation factors may contribute to the development of urologic complications (5). However, the incidence of urological complications after kidney transplantations is not known, nor their effect on graft survival (6, 7).

Patients with a urinary anastomotic complication have significantly more hospitalizations in first year after transplantation. The top readmission codes suggest that patient specific morbidity is directly related to the anastomotic complication and graft dysfunction. Fluid balance abnormalities develop, manifesting as fluid overload and increased acute cardiac events. Urinary tract and non-UTI infectious complications are also significantly increased in this patient population for unknown reasons. Acute renal failure is almost 2.5 times more likely to develop in patients with urinary anastomotic complications. Urinary leakage from ureteral anastomosis can lead to increased patient morbidity and costs (6).

Urologic complications of kidney transplantation have significantly decreased during the recent years. It seems that the most important factor for this decrease is firstly due to an improvement in immunosuppressive regimens and reduction in their use, and secondly due to technical improvements and the skillfulness of kidney surgeons (8, 9). The most common and important postoperative urologic complications after kidney transplantation are urinary fistula and then ureteral stricture (10, 11).

The association between the complication occurrence and the surgical technique has been evaluated in several studies (12-14). One of the most important factors influencing surgical technique is ureteral blood supply and ischemia in transplanted ureteral tissue. In some studies, ureteral length has been demonstrated as an effective factor in distal ureteral blood supply. However, other factors have also been proposed as factors resulting in urologic complications.

## 2. Objectives

Regarding increased rate of kidney transplantation during the past decades, this study was performed to evaluate the occurrence of long-term and short-term complications after the kidney transplantation and their association with the length of transplanted ureter. Although some effective factors have been previously evaluated in past studies, the role of ureteral length has not been evaluated so far.

## 3. Patients and Methods

### 3.1. Study Population

Between January 2008 and January 2011, 395 patients undergoing kidney transplantation in the Baqiyatallah and Moddares transplant centers of Tehran, Iran, were enrolled in the study. The kidney transplant recipients recruited at different times after kidney transplantation discharge.

### 3.2. Ethics

The protocol of this study was approved by the ethics committee of Baqiyatallah university of medical science, faculty of medicine.

### 3.3. Surgery Technique

Several operating surgeons were involved, of consultant or specialist registrar grade. In all but three procedures a Leadbetter-Politano ureteroneocystostomy was used, involving the passage of the ureter through a submucosal tunnel fashioned via a separate anterior cystostomy. Two cases required primary ureteroureterostomy for a short donor ureter. In one procedure the ureter was implanted into a bladder caecoplasty. Ureteric stents were used rarely. A Foley catheter was used to drain the bladders of all patients for at least 5 days after the operation. Daily serum biochemistry was combined with careful clinical observation to monitor graft function then image all grafts by ultrasonography soon after the operation, usually in the first or second day, to detect early signs of vascular or urological complications. This succeeds the former practice of imaging only in those with suspected graft dysfunction.

### 3.4. Immunosuppressant Regimen

Cyclosporine (CsA) was taken orally as a basic drug for immunosuppression in kidney transplant patients; Mycophenolate Mofetil/Azathioprine and Prednisolone were also used. To prevent rejection, high doses of CsA were routinely started and it was reduced subsequently. The dosage was initially determined by the body weight of the individuals and subsequently by the CsA blood Levels. Dosages also varied from one individual to another depending on the patient's ability to withstand organ rejection. Our target for cyclosporine through level (C0) blood levels were 200 to 300 ng/mL in months one to three after transplantation, 100 to 250 ng/mL in 4 to 12 months and 100 to 150 ng/mL in more than 1 year after the transplantation; though the 2-hour post dose level of CsA (C2) optimum levels of 800 to 1000 ng/mL 1 to 3 months after transplantation and C2 targets of 400 to 600 ng/mL for following months.

### 3.5. Variables

Routine laboratory investigations included creatinine (Cr). The biochemical analyzes were performed by the unique laboratory using automatic systems with modification of the kinetic Jaffe reaction (Cr).

### 3.6. Definitions

Graft loss to chronic rejection was defined by pathologic findings, on biopsy or at nephrectomy, that were consistent with chronic rejection. The concept of defining death with function as graft loss originated with the recognition that many post-transplant deaths (with function) were due to infection secondary to the surgery and immunosuppressive regimens; more recently, it has been noted that immunosuppression-related hypertension, hyperlipidemia, and diabetes affect post-transplant mortality.

### 3.7. Statistical Analysis

The SPSS version 17.0 for Windows was used in all the analyses. Quantitative variables were expressed as mean ± standard deviation (SD), while qualitative variables were shown by number and percentage. The kolmogorov-simirnov test showed that ureteral length distributed normally; hence, T test analysis was used to study correlations between complication and ureteral length less and more than 5.5 cm, for assessing highest specific and sensitive ureteral length we used ROC Curve estimation and we used Kaplan-Meier survival analysis. In all analyses P < 0.05 was considered significant with 95% confidence Interval.

## 4. Results

### 4.1. Demographical Setting

A total of 395 patients with the mean age of 37 years (range, 18 to 68 years) were enrolled in the study, Twenty six graft lost during the follow-up period. All deaths were after the second postoperative month and because of medical and nonsurgical causes. Mean ages of the donors and recipients were 27 ± 4 versus 37 ± 13 years. Baseline characteristic are shown in Table 1. 

**Table 1.** Baseline Characterizations of Kidney Transplant Recipients

Variable	
**Donor Age, (mean ± SD)**	27.26 ± 4.86
**Male Recipients, No.(%)**	267 (66.8)
**Recipient’s age, (mean ± SD)**	37.42 ± 13.37
**Ureteral Length, (mean ± SD)**	9.10 ± 1.33
**Left Side Transplantation, No.(%)**	299 (75.7)
**Baseline Creatinine, (mean ± SD)**	1.45 ± 1.27
**Last Creatinine, (mean ± SD)**	1.82 ± 1.28
**Operation Time, (mean ± SD)**	102.67 ± 18.43
**Follow up Time, (mean ± SD)**	47.14 ± 8.12

The Mean length of the transplanted ureter was 9.09 ± 1.31 cm versus 9.25 ± 1.53 cm, respectively (P = 0.50). The Mean serum creatinine level at discharge was 1.35 ± 1.09 mg/dL versus 2.40 ± 2.21 mg/dL, respectively (P = 0.008). The Mean hospital staying was 16.58 ± 8.57 days versus 27.27 ± 22.81 days, respectively (P = 0.008). 

### 4.2. Surgical Complications

Urinary stenosis was seen in 6 patients (1.5%) and urinary leakage was seen in 4 (1%) patients, other complications are shown in Table 2. 

**Table 2.** Complications After Kidney Transplantation

Variable	
**Urinary Stenosis, No.(%)**	6 (1.5)
**Urinary Leakage, No.(%)**	4 (1)
**Number of Hospitalization Days, (mean ± SD)**	17.55 ± 11.07
**Graft Loss, No.(%)**	26 (6.6)
**Graft Loss Time, (mean ± SD)**	23.50 ± 13.76
**Deaths, No.(%)**	10 (2.5)
**Deaths Time, (mean ± SD)**	30.91 ± 10.64

### 4.3. Post-transplant Course

**Figure 1.**
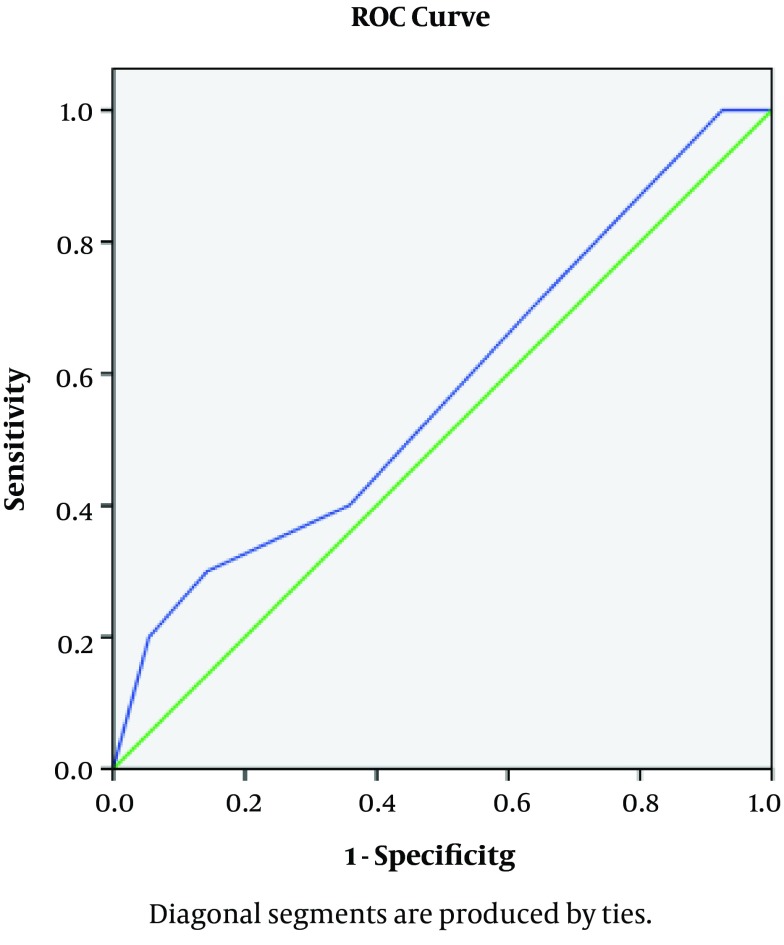
Roc Curve of Ureteral Length and Graft Loss

The Mean follow-up period was 47 months (range, 3 to 54 months). After transplantation by ROC curve we found the ureter size of 5.5 with best specificity and sensitivity for complication (Figure 1).

The patients were divided into 2 groups regarding the ureteral length. The complication rate was not significantly different between these groups (P = 0.67).

**Figure 2.**
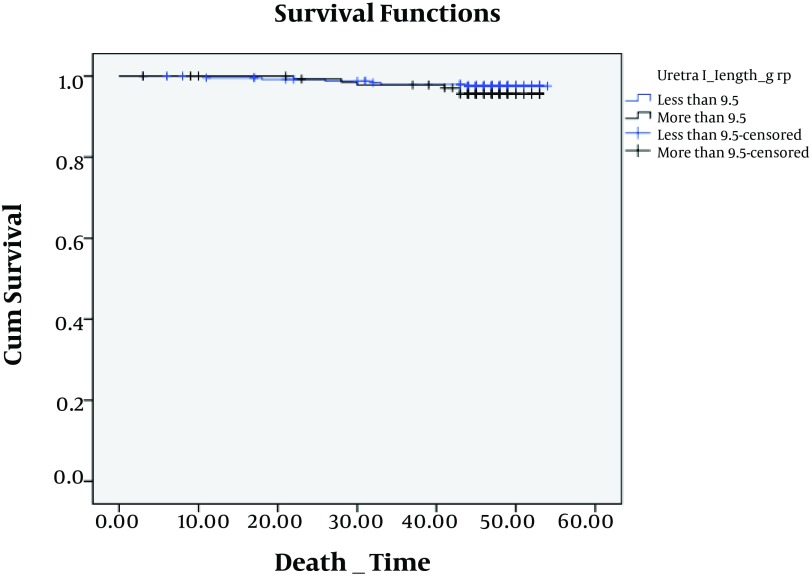
Kaplan-Meier Survival of Patient With Ureteral Length Less and More than 9.5 cm

**Figure 3.**
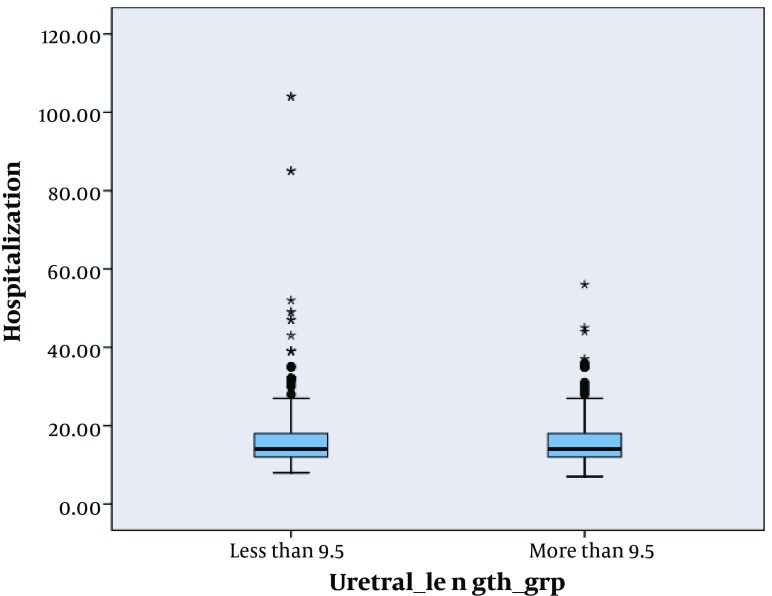
Box Plot of Hospitalization Difference Regarding Ureteral Length Less and More Than 9.5 cm

**Figure 4.**
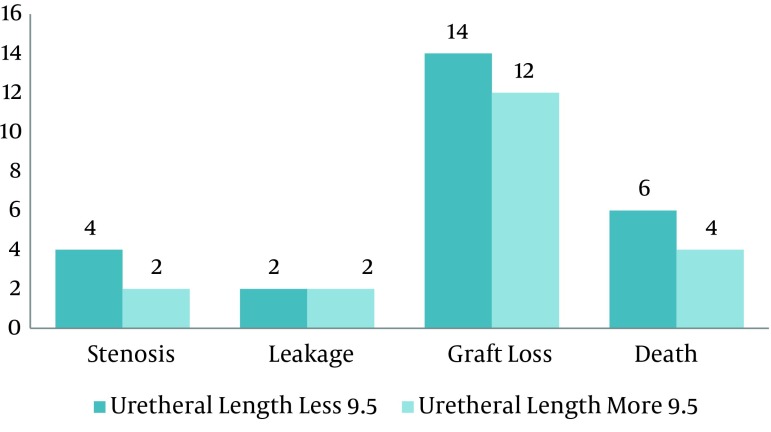
Bar Chart Shows Complication After Kidney Transplantation in Ureteral Length Less and more Than 9.5 cm

We analyzed survival for graft, time of hospitalization and complication after surgery by means of this length. We found that there were no significant difference among complications (P = 0.25), hospitalization (P = 0.31) and survival (P = 0.84) at this cut off (Figures 2, 3 and 4).

## 5. Discussion

Surgical complications remain a significant clinical problem after renal transplantation. Ureteral obstruction following transplantation is not uncommon. Persistent obstruction of the ureterovesical anastomosis is the most common urologic complication. Obstruction occurring beyond the first postoperative month remains frequent (2-7.5%) and mostly related to ureteral stenosis. The overall incidence of urologic complications in our series was low, which was comparable to those reported by other major centers (15-18).

Urologic complications associated with the ureterovesical anastomosis after transplantation may cause graft loss and mortality (19). Complications vary from around 20% to less than 5% (15, 16). At many transplantation centers, surgeons have adopted new suture techniques. Several preventive measures have been added to this technique to prevent urologic complications (16).

For avoiding anastomotic stricture, kinking and urinary leakage, some surgeons routinely anastomose ureter to bladder over an ureteral stent (20, 21).

Our major finding was that ureteral length is not related to kidney transplantation complication although it showed that ischemia is the most common cause of distal ureteral stricture formation often involving the ureterovesical junction. The association between the ureteral complications and the length of transplanted ureter has always been in attention due to its probable role in ischemia of the transplanted ureteral tissue ant it is challenging.

According to our findings, the ureteral length was not significantly different in the patients with and without complications. This compromised blood supply can be due to problems in operative technique during harvesting or high dose of immunosuppression (22). Some experience with rat showed that preservation of adequate blood supply to the ureter in renal transplantation provides more consistent results and lessens the risk of unnecessary animal loss (23). Other risk factors for ureteral stenosis include (24) ischemia, rejection, calculi, fungal ball, clots, technical error, fluid compression (25-31).

Several studies have been performed in this regard; however, demographic and anatomic factors have not been studied in none of them. Benoit and colleagues studied 430 patients with kidney transplantation within 5 years and showed that urologic complication rate was about 12% in ureteroureterostomy while it was about 6.7% in ureteroneocystostomy. It was also demonstrated that the major factor in occurrence of ureteral complications is transplanted ureteral length rather than the type of ureteral anastomosis; i.e., the longer the ureter, the more the urologic complications (12). McDonald and coworkers showed that ureteroneocystostomy accompanies by a decrease in the complication rates, and one reason for this decrease is the need for a shorter ureter. Also, using this technique, catheter cystostomy was not necessary and antireflux mechanism was preserved (14). Actually, in both studies mentioned, ureteral blood supply has been regarded as a dependent variable to the ureteral length. They only concluded the study regarding the ureter-to-bladder anastomosis technique without the measurement of the ureteral length.

Khavli and colleagues studied these two subjects separately on animal models for the first time and evaluated the transplanted ureteral blood supply regarding the technique used for detachment of the ureter from adjacent tissues. They concluded that the preservation of the gonadal artery causes in significant decrease in the occurrence of ureteral complications (23). Alfani and coworkers showed that the obstruction of transplanted ureter may be an early or late complication, and usually does not cause kidney colic because of the lack of innervation to the transplanted kidney. They showed that the obstruction usually occurs at the end of the distal ureter. They proposed the probable cause of ischemia for this and emphasized that the effective factor in obstruction of transplanted ureter is the involvement of pelvis and ureter and fibrosis of the adjacent tissues. They concluded that the ureteral obstruction hardly happens without hydronephrosis (32). Cecka and colleagues defined the damage during the surgery and distal ureter ischemia as the most important effective factors on urinary fistula (10, 33). Thomalla showed that urinary fistula may affect transplantation rejection because of inappropriate detachment of the donor ureter, stretch on transplanted ureter due to the shortness of the detached ureter, rupture of the ureter or the kidney pelvis due to severe obstruction, deep and invasive infection of the wound, ureter destruction because of the pelvic infection, and/or slow blood supply. He concluded that the ureteral length was an effective factor only in case of being short (because of the stretch on the ureter) (34).

 In our study lower complication versus 9% in Benoit’s study is probably due to improvement in surgical techniques, increased skillfulness of the surgeons, development in using immunosuppressive drugs after the transplantation. The authors emphasized that the urologic complications after kidney transplantation are affected by the position of the transplanted kidney, kidney positioning and the type of ureteral anastomosis. The difference in the results between their study and ours is probably due to using ureteroureterostomy by them which accompanies with high complication rates. But after changing their method from ureteroureterostomy to ureteroneocystostomy, the complication rate decreased significantly, and this resulted in less accuracy in their study.

Conclusion: According to our findings, it can be concluded that the length of the transplanted ureter does not have any association with the frequency of urologic complication occurrence. It seems that improvement in surgical techniques, surgeons’ skillfulness and development in using immunosuppressive drugs are the factors decreasing this rate. We can conclude that not considering the ureteral length is a safe limit in kidney transplantation surgeries, and ureters between 7–12 m can be anastomosed safely without any effect on urologic complications rate.
